# Illumina next-generation sequencing reveals the mitochondrial genome of *Ducetia japonica* (Orthoptera: Tettigoniidae)

**DOI:** 10.1080/23802359.2016.1168717

**Published:** 2016-06-20

**Authors:** Bei Guan, Huifang Guo, Zhijun Zhou

**Affiliations:** College of Life Sciences, Hebei University, Baoding, China

**Keywords:** *Ducetia japonica*, Illumina next-generation sequencing, mitochondrial genome, Orthoptera

## Abstract

Using Illumina next-generation sequencing (NGS), the complete mitochondrial genome (mitogenome) of *Ducetia japonica* was sequenced in the present study. The mitogenome of *D. japonica* (Genbank accession no. KU885974) is 16,276 bp in size, had the typical invertebrate mitochondrial gene arrangement and containing 13 protein-coding genes (PCGs), 22 transfer RNA genes (tRNA), 2 ribosomal RNA genes (rRNA) and a control region. Except for the control region (868 bp), one novel larger intergenic (616 bp) was found between *nad2* and *trnW*. Phylogenetic results unambiguously support the monophyly of Phaneropterinae, although the gene order of two *Sinochlora* species different with other Tettigoniidae species. Using Illumina NGS platforms for mitogenome sequencing will provide rather essential and important DNA molecular data for the further phylogenetic analysis across major ensiferan lineages.

*Ducetia japonica* is widely distributed throughout China, and its type locality is Asia-Temperate, Eastern Asia, Japan. Next-generation sequencing (NGS) is an effective method for mitochondrial genome (mtDNA) sequencing and heteroplasmy detection (Dames et al. 2015). NGS has been presented as a valuable means to collect the mitochondrial genome sequences of parasitic worms (Jex et al. 2010), nematodes (Webb & Rosenthal 2011) and fishes (Chen et al. 2015). In this study, we used Illumina NGS technology to sequence the mitogenomes of *D. japonica*, with the purpose to offer the genetic information for developing new DNA markers and evolutionary analysis for *D. japonica*.

The specimen was obtained from Diaoluoshan National Forest Park (18.78 N, 109.86 E), Hainan, China, and stored in Hebei University (No. ZZJ173). The total genomic DNA was extracted from the leg muscle tissue of a single adult male specimen using the TIANamp. A total of 1,845,072,900 bp clean data were generated on the Illumina HiSeq2000 at BGI-Shenzhen, China. De novo assembly of *D. japonica* mitogenome using SOAPdenovo-Trans (-K 71) (Xie et al. 2014), and annotated using custom Perl script described by (Zhou et al. 2013) with a reference data base of 774 arthropod mitogenomes (Tang et al. 2014).

The mitogenome of *D. japonica* (Genbank accession no. KU885974) is 16,276 bp in size and contains of 13 protein-coding genes (PCGs), 22 tRNA genes, two rRNA genes and a control region (Table 1). In previous study, we reported the *Elimaea cheni* mitogenome from subfamily Phaneropterinae, and found it with the inferred ancestral gene order for insect (Zhou et al. 2010). However, a recent study identified one novel gene order rearrangement “rrnS-trnI-trnM-nad2-CR-trnQ-trnW” in two *Sinochlora* species from Phaneropterinae (Liu et al. 2013). The gene order rearrangement of *D. japonica* mitogenome is identical with *E. cheni*, and no rearrangements, duplications, or deletions of any genes (Boore 1999).

**Table 1.** Characteristics of the *D. japonica* mitochondrial genome.

Gene/region	Strand	Position	Size (bp)	Start/stop codon	Gene/region	Strand	Position	Size (bp)	Start/stop codon
*Ile*	J	1–68	68		*Asn*	J	6668–6732	65	
*Gln*	N	66–134	69		*Ser^AGN^*	J	6732–6797	66	
*Met*	J	149–215	67		*Glu*	J	6798–6864	67	
*nad2*	J	216–1235	1020	ATG-TAA	*Phe*	N	6866–6930	65	
*Trp*	J	1852–1917	66		*nad5*	N	6931–8662	1732	ATT-T
*Cys*	N	1917–1981	65		*His*	N	8663–8725	63	
*Tyr*	N	1982–2049	68		*nad4*	N	8726–10064	1339	ATG-T
*cox1*	J	2042–3581	1540	ATT-T	*nad4L*	N	10058–10354	297	ATG-TAA
*Leu^UUR^*	J	3582–3648	67		*Thr*	J	10360–10424	65	
*cox2*	J	3652–4342	691	ATG-T	*Pro*	N	10424–10488	65	
*Lys*	J	4343–4412	70		*nad6*	J	10490–11011	522	ATC-TAA
*Asp*	J	4412–4478	67		*Cytb*	J	11011–1150	1140	ATG-TAG
*atp8*	J	4479–4646	168	ATT-TAA	*Ser^UCN^*	J	12149–12215	67	
*atp6*	J	4640–5314	675	ATG-TAA	*nad1*	N	12232–13179	948	ATT-TAG
*cox3*	J	5314–6102	789	ATG-TAA	*Leu^CUN^*	N	13183–13245	63	
*Gly*	J	6104–6168	65		*rrnL*	N	13246–14553	1308	
*nad3*	J	6169–6522	354	ATT-TAA	*Val*	N	14554–14622	69	
*Ala*	J	6527–6595	69		*rrnS*	N	14623–15408	786	
*Arg*	J	6595–6660	66		Control region		14409–16276	868	

The size of PCGs in *D. japonica* mitogenome is similar to their corresponding orthologs in other ensiferan species. The start codon ATN, such as ATC for *nad6*, ATT for *cox1*, *nad1* and *nad5*, ATG for remaining PCGs, was found in all PCGs. There are three types stop codon was found as following: TAA (*atp6*, *atp8*, *cox3*, *nad2*, *nad3*, *nad4L* and *nad6*), TAG (*cytb*, *nad1*), and T (*cox1*, *cox2*, *nad4* and *nad5*). The size of 22 tRNA genes vary from 63 to 70 bp, and all tRNA genes could be folded into the typical cloverleaf secondary structure except for *trnS^AGN^*. The size of *rrnL* and *rrnS* genes, located between *trnL* gene and control region, and was separated by *trnV* gene, are determined to be 1308 bp and 786 bp, respectively. Except for the control region (868 bp), one novel larger intergenic (616 bp) was found between *nad2* and *trnW*.

Phylogenetic relationships of Tettigoniidae were determined based on 13 PCGs and two rRNA genes sequences by Bayesian Inference (BI) implemented in MrBayes 3.1.2 (MrBayes Inc., Tallahassee, FL) (Figure 1). Our phylogenetic results unambiguously support the monophyly of Phaneropterinae, although the gene order of two *Sinochlora* species different with other Tettigoniidae species (Liu et al. 2013).

**Figure 1.**
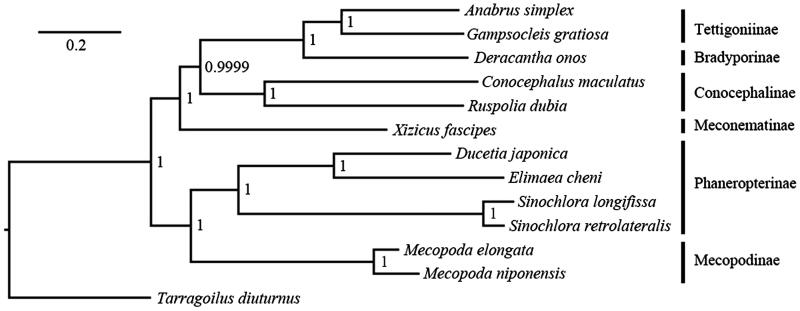
Phylogenetic relationships of Tettigoniidae inferred from 13 PCGs and two rRNA genes by Bayesian Inference. BI analysis was carried out with unlinked partitions, appropriate models for each partition using four independent Markov chains, 1,500,000 generations, sampling every 100 generations with a burn-in of 25%. Genbank accession numbers for each species are the following: *Anabrus simplex* (EF373911), *Conocephalus maculatus* (HQ711931), *Deracantha onos*(EU137664), *Elimaea cheni* (GU323362), *Gampsocleis gratiosa* (EU527333), *Kuwayamaea brachyptera* (KT345950), *Mecopoda elongate* (JQ917910), *Mecopoda niponensis* (JQ917909), *Sinochlora longifissa* (KC467055), *Sinochlora retrolateralis*(KC467056), *Xizicus fascipes* (JQ326212), *Ruspolia dubia* (EF583824), and outgroup *Tarragoilus diuturnus* (JQ999995).
